# Diagnostic Accuracy of Combined Fine-Needle Aspiration Biopsy and Ultrasonography for Malignancy in Bethesda III–IV Thyroid Nodules

**DOI:** 10.3390/jcm15155853

**Published:** 2026-07-27

**Authors:** Sinan Aslan, Şebnem Çimen

**Affiliations:** 1 Department of General Surgery, Mersin City Training and Research Hospital, 33240 Mersin, Türkiye; drsinanaslan84@gmail.com; 2 Department of General Surgery, Akyurt State Hospital, 06750 Ankara, Türkiye

**Keywords:** thyroid nodules, ultrasonographic features, fine-needle aspiration biopsy, Bethesda 3, Bethesda 4, AUS

## Abstract

**Background**: Management of Bethesda III–IV thyroid nodules remains challenging because of diagnostic uncertainty and variable malignancy risk. This study evaluated the diagnostic performance of fine-needle aspiration biopsy (FNAB) and ultrasonographic (USG) features separately and in combination. **Methods**: This retrospective cohort study included 1800 patients with Bethesda III–IV thyroid nodules who underwent thyroidectomy between 2017 and 2025. FNAB results and USG features, including hypoechogenicity, microcalcifications, and irregular margins, were analyzed. Logistic regression and ROC curve analyses were performed to determine predictors of malignancy and diagnostic performance. **Results**: The overall malignancy rate was 25%. Malignancy rates were 19.1% in Bethesda III and 37.4% in Bethesda IV nodules (*p* < 0.001). In multivariable analysis, Bethesda IV category, microcalcifications, irregular margins and hypoechogenicity were independent predictors of malignancy (all *p* < 0.001). FNAB and USG alone showed limited discrimination (AUC 0.60 and 0.61, respectively), whereas the combined model demonstrated significantly improved performance (AUC 0.78). The combined model achieved 82.4% sensitivity, 70.1% specificity and 92.1% negative predictive value. **Conclusions**: USG features provide significant additional diagnostic value beyond cytology in Bethesda III–IV thyroid nodules. Combining USG findings with FNAB improves risk stratification and may support clinical decision-making in indeterminate thyroid nodules.

## 1. Introduction

Thyroid nodules are commonly encountered in the general population and their detection rates have increased significantly with the widespread use of high-resolution non-invasive imaging modalities such as USG [1,2,3]. Although the majority of nodules are benign, accurate determination of malignancy risk constitutes the cornerstone of clinical management [4,5]. Therefore, reliable and clinically applicable preoperative risk stratification strategies are needed [6,7].

FNAB remains the primary diagnostic method for the evaluation of thyroid nodules and is widely standardized using the Bethesda classification system [8,9]. However, Bethesda category III and Bethesda category IV are considered cytologically indeterminate thyroid nodules and remain a diagnostic challenge in clinical practice. Particularly in the Bethesda III group, patients are frequently referred for repeat FNAB procedures due to the indeterminate nature of the initial cytology; however, this approach does not always provide definitive diagnostic clarification [10,11,12]. Reported malignancy rates in these categories range approximately from 13% to 30% for Bethesda III and from 23% to 34% for Bethesda IV nodules [10], although these rates may vary depending on patient selection and reclassification criteria [13,14,15]. This situation represents a significant clinical challenge that may lead both to unnecessary surgical interventions and delayed diagnosis of malignancy.

USG not only provides morphological assessment in the evaluation of thyroid nodules but also serves as a fundamental tool playing a decisive role in clinical risk stratification. Numerous studies have demonstrated that USG features such as hypoechogenicity, microcalcifications, irregular margins and a taller-than-wide shape are associated with malignancy [16,17]. Based on these findings, risk stratification systems such as ATA, ACR-TIRADS and EU-TIRADS have been developed and widely adopted in clinical practice [6,7,18,19]. Nevertheless, the diagnostic performance of these systems may vary and they may fail to achieve sufficient discriminative power when used alone, particularly in cytologically indeterminate nodules [20,21].

In the current literature, the association of FNAB cytology and USG findings with malignancy has often been evaluated separately or reported descriptively. However, studies quantitatively assessing the diagnostic contribution of combining these two modalities, particularly those demonstrating the incremental value of USG features beyond cytological classification through model-based analyses, remain limited [22]. Consequently, the potential benefits of a multimodal approach in clinical decision-making processes have not been sufficiently elucidated.

In this context, more comprehensive and data-driven approaches are needed to reduce diagnostic uncertainty in Bethesda III–IV thyroid nodules. Integration of USG features with cytological data may not only improve diagnostic accuracy but also enhance patient-based risk stratification, thereby enabling more rational surgical decision-making.

The aim of this study was to evaluate the diagnostic performance of FNAB and USG findings, both separately and in combination, for predicting malignancy in Bethesda III–IV thyroid nodules and additionally to quantitatively determine the incremental contribution of USG features beyond cytological classification through model-based analyses.

## 2. Materials and Methods

### 2.1. Study Design and Setting

This study was planned in a retrospective cohort design conducted at the General Surgery Clinic of Mersin City Training and Research Hospital. In this analysis carried out at a tertiary referral center, it was aimed to evaluate both separately and together the diagnostic performance of preoperative FNAB findings and USG features in predicting malignancy in Bethesda category III–IV thyroid nodules. The study was conducted in accordance with the principles of the Declaration of Helsinki, and the STROBE guideline recommended for observational studies was taken into consideration during the reporting process.

### 2.2. Ethical Approval

The required ethical approval for the study was obtained from the Clinical Research Ethics Committee of Mersin City Training and Research Hospital (25 March 2026, decision no: 2026/160). Due to the retrospective nature of the study and anonymization of the data, the requirement for individual informed consent was waived by the ethics committee.

### 2.3. Patient Population and Selection Process

A total of 2100 patients who underwent thyroidectomy between 1 January 2017 and 31 December 2025 were retrospectively evaluated. After the application of predefined exclusion criteria, 1800 patients constituted the final analysis group. Patients whose preoperative FNAB results were reported as Bethesda category III or IV, who had adequate preoperative USG evaluation and who had a definitive postoperative histopathological diagnosis were included in the study. Cases with missing FNAB results, inadequate ultrasonography records, previous thyroid surgery or unavailable histopathological diagnosis were excluded from the study. With this approach, it was aimed to obtain a comparable patient group with complete cytological and imaging data.

### 2.4. Data Collection and Variables

Demographic characteristics, nodule size and histopathological results of the patients were obtained through the hospital information management system and anonymized before analysis. FNAB results were evaluated according to the Bethesda System for Reporting Thyroid Cytopathology, and only category III and IV nodules were included in the study. In USG examinations, hypoechogenicity, presence of microcalcifications and irregular margins, which are the parameters most strongly associated with malignancy, were analyzed. Hypoechogenicity was defined as echogenicity lower than that of the surrounding thyroid parenchyma, microcalcifications as punctate echogenic foci measuring ≤1 mm without posterior acoustic shadowing and irregular margins as spiculated, microlobulated or ill-defined lesion borders. All ultrasonographic evaluations were performed by experienced radiologists routinely working at the center and analyses were conducted based on standard reports.

### 2.5. Outcome Measure

The primary endpoint of the study was defined as confirmation of the presence of malignancy as a result of histopathological examination of the surgical specimen. Accordingly, patients were divided into benign and malignant groups.

### 2.6. Statistical Analysis

All statistical analyses were performed using IBM SPSS Statistics software (version 29.0; IBM Corp., Armonk, NY, USA). Continuous variables were expressed as mean ± standard deviation, while categorical variables were expressed as number and percentage. An independent samples *t*-test was used for continuous variables and a chi-square test for categorical variables in intergroup comparisons. Cases with missing data were excluded from the analysis, and analyses were conducted on the complete dataset. In all tests, a two-sided *p* value below 0.05 was considered statistically significant.

### 2.7. Univariable Analysis

Separate logistic regression analyses were applied for each variable in order to evaluate the relationship between USG features and malignancy and the results were reported with odds ratios and 95% confidence intervals. This analysis was used to demonstrate the independent effects of the variables for preliminary evaluation purposes.

### 2.8. Multivariable Logistic Regression Analysis

A multivariable logistic regression model was established to determine independent predictors of malignancy. Hypoechogenicity, microcalcification and irregular margins were included in the model together with Bethesda category. The possibility of multicollinearity in the model was evaluated using the variance inflation factor and no significant collinearity was detected. While model fit was examined with the Hosmer–Lemeshow test, the level of explanatory power was expressed using the Nagelkerke R^2^ value.

### 2.9. Diagnostic Performance Analysis

The discriminative performance of FNAB, USG and the model in which these two methods were used together was evaluated with receiver operating characteristic (ROC) curve analysis. The area under the curve (AUC) was calculated for each model and 95% confidence intervals were reported. In addition, sensitivity, specificity, positive predictive value, negative predictive value and overall accuracy rate were calculated and clinically compared.

### 2.10. Model Development and Incremental Value Analysis

Two nested models were created in order to evaluate the additional contribution of USG findings to cytology. While the first model included only the Bethesda category, USG features were also added in the second model. The models were compared using −2 log likelihood and Akaike information criterion and improvement in model fit was evaluated with the likelihood ratio test. In addition, the AUC difference was calculated to demonstrate the change in discriminative power.

### 2.11. Subgroup Analysis

The performance of the combined model was evaluated separately in Bethesda III and Bethesda IV subgroups. ROC curves were generated for each subgroup, AUC values were calculated and the performance of the model in different cytological risk groups was examined.

## 3. Results

**Figure 1:**
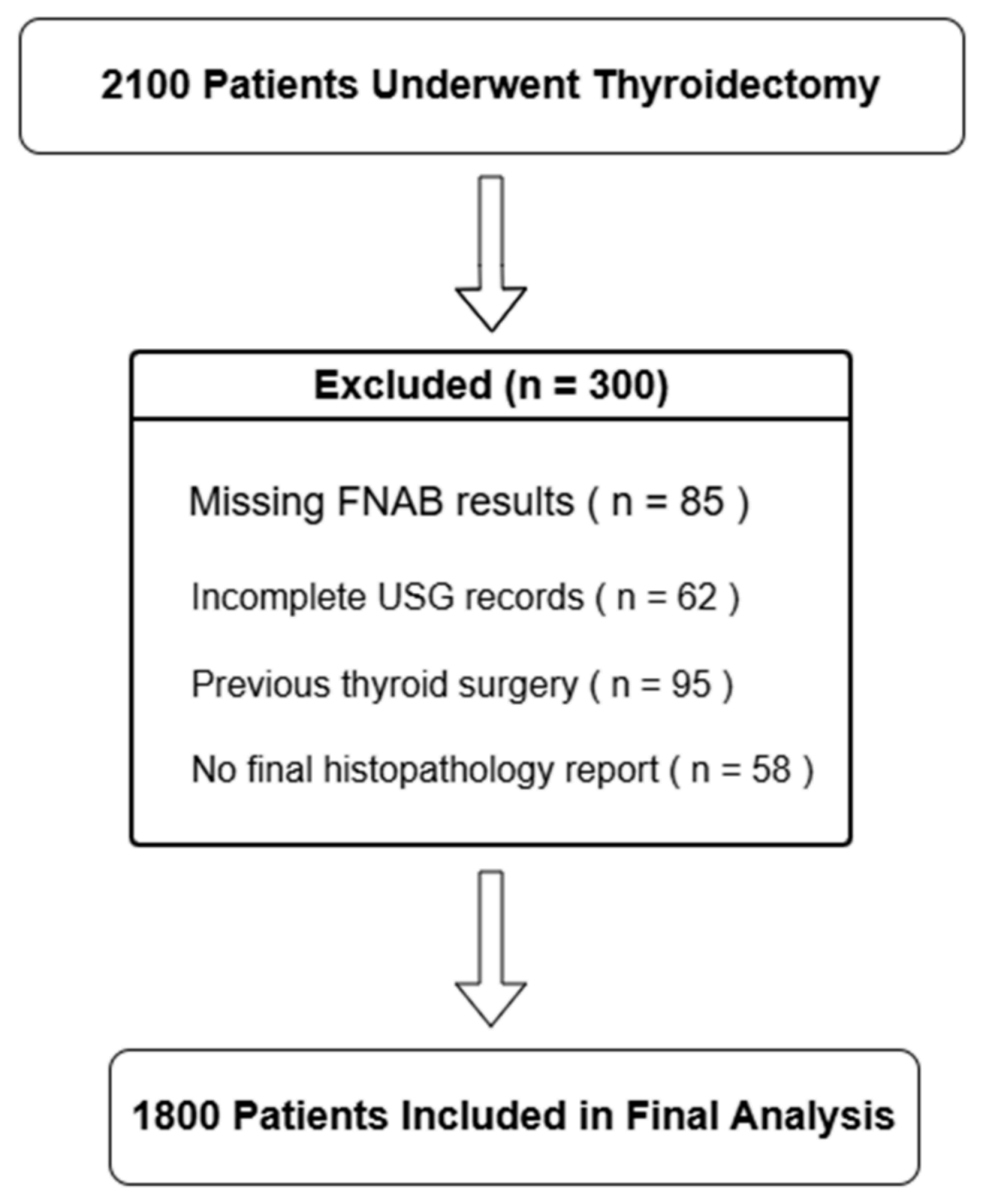
Study flow diagram. Study flow diagram demonstrating the screening and inclusion process. Among 2100 consecutive patients undergoing thyroidectomy, 300 were excluded based on predefined criteria (missing FNAB results, incomplete USG records, prior thyroid surgery or unavailable final histopathology). The remaining 1800 patients constituted the final analytic cohort.

A total of 2100 patients who underwent thyroidectomy during the study period were retrospectively evaluated and after the application of predefined exclusion criteria, 1800 patients constituted the final analysis group. A substantial proportion of the 300 excluded patients consisted of cases with missing or inaccessible FNAB results, inadequate USG records, a history of previous thyroid surgery or unavailable histopathological data. The patient selection process is presented in detail in Figure 1. Among the 1800 patients included in the final analysis group, malignancy was detected in 450 patients (25%), whereas benign histopathological results were obtained in 1350 patients (75%).

The demographic and clinical characteristics of the patients are shown in detail in Table 1. The mean age of the entire population was calculated as 46.8 ± 12.4 years. The mean age of patients in the malignant group was significantly lower compared to the benign group (45.2 ± 11.9 years vs. 47.3 ± 12.6 years, *p* = 0.002). When gender distribution was evaluated, 73.8% of the entire population were female; this rate was 74.4% in the benign group and 71.8% in the malignant group, and no statistically significant difference was found between the groups (*p* = 0.28). While the mean nodule size was 21.6 ± 8.9 mm in the entire group, it was measured as 22.4 ± 9.1 mm in the benign group and 19.1 ± 7.8 mm in the malignant group; nodule size was found to be significantly smaller in the malignant group (*p* < 0.001).

When Bethesda category distribution was evaluated, 67.9% of all cases were found to be in Bethesda III and 32.1% in Bethesda IV category. While the rate of Bethesda III was 73.3% in the benign group, this rate was found to be 52.0% in the malignant group. In contrast, the rate of Bethesda IV nodules was 26.7% in the benign group and 48.0% in the malignant group and this difference was statistically significant (*p* < 0.001). Malignancy rates according to Bethesda categories are presented in detail in Table 2. Accordingly, the malignancy rate was calculated as 19.1% (234/1223) in Bethesda III nodules, whereas this rate was found to be 37.4% (216/577) in Bethesda IV nodules (*p* < 0.001).

The distribution of USG features and their relationship with malignancy are shown in Table 1 and Table 3. Hypoechogenicity was detected in 42.7% of the entire population, whereas it was observed in 37.6% of the benign group and 58.0% of the malignant group (*p* < 0.001). The presence of microcalcifications was observed at a rate of 35.7% overall, whereas it was detected in 27.5% in the benign group and 60.4% in the malignant group (*p* < 0.001). Similarly, irregular margins were found at a rate of 33.5% in the benign group and 60.4% in the malignant group (*p* < 0.001). In univariable logistic regression analysis, hypoechogenicity (OR 2.30, 95% CI 1.87–2.83, *p* < 0.001), microcalcification (OR 3.99, 95% CI 3.23–4.93, *p* < 0.001) and irregular margins (OR 3.02, 95% CI 2.45–3.72, *p* < 0.001) were found to be significantly associated with malignancy.

The results of the multivariable logistic regression analysis are presented in Table 4. All variables included in the model were found to be independently associated with malignancy. Bethesda IV category was associated with a higher risk of malignancy compared to Bethesda III (aOR 2.11, 95% CI 1.65–2.70, *p* < 0.001). Among USG parameters, microcalcification was identified as the strongest independent predictor (aOR 2.48, 95% CI 1.89–3.25, *p* < 0.001). Irregular margins (aOR 1.93, 95% CI 1.46–2.54, *p* < 0.001) and hypoechogenicity (aOR 1.72, 95% CI 1.34–2.21, *p* < 0.001) were also identified as significant independent variables. When model calibration was evaluated with the Hosmer–Lemeshow test, model fit was found to be adequate (*p* = 0.42). The explanatory level of the model was calculated as 0.26 with the Nagelkerke R^2^ value and the overall accuracy of the model was found to be 72.3%.

**Figure 2:**
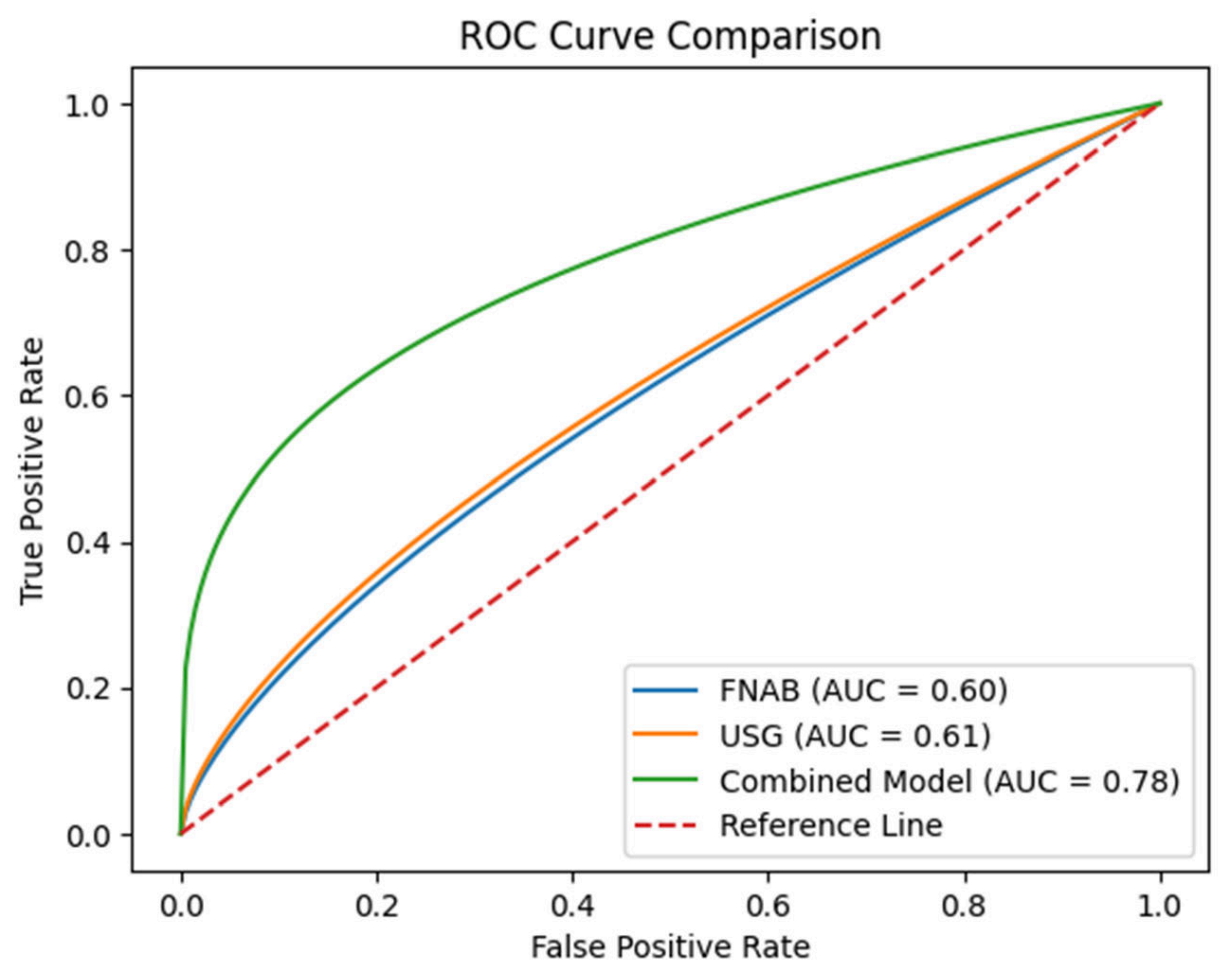
ROC curves of FNAB, USG, and the combined model for predicting malignancy. ROC curves comparing the diagnostic performance of FNAB alone, USG alone and the combined multivariable model incorporating Bethesda category and USG features for predicting malignancy in Bethesda III–IV thyroid nodules. The combined model demonstrated superior discriminative ability (AUC = 0.78) compared with FNAB alone (AUC = 0.60) and USG alone (AUC = 0.61).

The results of the diagnostic performance analysis are presented in Table 5 and Figure 2. When FNAB was evaluated alone, sensitivity was calculated as 76.0%, specificity as 43.4%, positive predictive value as 30.9%, negative predictive value as 84.4%, and overall accuracy as 51.6%. When USG findings were evaluated alone, sensitivity was found to be 58.0%, specificity 62.4%, positive predictive value 33.9%, negative predictive value 81.7% and accuracy 61.3%. In the combined model in which FNAB and USG findings were evaluated together, sensitivity was calculated as 82.4%, specificity as 70.1%, positive predictive value as 47.6%, negative predictive value as 92.1% and overall accuracy as 74.8%. In ROC curve analysis, the AUC value was found to be 0.60 (95% CI 0.57–0.63) for FNAB, 0.61 (95% CI 0.58–0.64) for USG and 0.78 (95% CI 0.75–0.81) for the combined model.

The findings of the subgroup analysis are presented in Table 6. In the Bethesda III group, the malignancy rate was calculated as 19.1%. In this group, the sensitivity of the combined model was 76.9%, specificity 74.8%, positive predictive value 39.8%, negative predictive value 93.8% and accuracy 75.3%. In ROC analysis, the AUC value was calculated as 0.74 (95% CI 0.70–0.78). In the Bethesda IV group, the malignancy rate was found to be 37.4%. In this group, the sensitivity of the combined model was calculated as 85.2%, specificity 68.1%, positive predictive value 58.3%, negative predictive value 88.9% and accuracy 73.4%. In ROC analysis, the AUC value was found to be 0.80 (95% CI 0.76–0.84).

The findings of the model comparison and incremental value analysis are presented in Table 7. In the model including only Bethesda category, the −2 log likelihood value was calculated as 2018.4 and the AIC value as 2022.4. In the model in which USG features were added, the −2 log likelihood value decreased to 1786.2, whereas the AIC value was found to be 1796.2. This improvement in model fit was found to be statistically significant (Δ−2LL = 232.2, *p* < 0.001). In addition, a marked increase in discriminative power was detected, and the AUC value was calculated to increase from 0.60 to 0.78 (ΔAUC = 0.18).

## 4. Discussion

In this study, the performance of FNAB and USG findings in predicting malignancy in Bethesda category III–IV thyroid nodules was evaluated separately and together. The obtained findings demonstrate that the combined use of cytological and USG data significantly increases diagnostic performance and provides a marked superiority, particularly in terms of discriminative power. These results support that current cytology-based approaches alone may not be sufficient and reinforce the necessity of multimodal evaluation.

The primary aim in the evaluation of thyroid nodules is to accurately determine malignancy risk in order to reduce unnecessary surgeries and identify malignant cases in a timely manner. Current guidelines such as ATA and ETA recommend the combined evaluation of both cytological and USG findings in risk stratification [6,7]. Nevertheless, there is no standardized algorithm regarding how this approach should be applied, particularly in cytologically indeterminate nodules. This study provides a quantitative evaluation aimed at filling this gap.

Bethesda category III and IV nodules constitute the most problematic group in terms of clinical management. It is known that the malignancy rates reported for these categories vary within a wide range in the literature [10,13,14,15]. This variability is influenced by patient selection, center experience, and reclassification processes such as NIFTP. In our study, the malignancy rate was found to be 19.1% in Bethesda III nodules and 37.4% in Bethesda IV nodules, and these values are consistent with the literature. In a Turkish single-center study by Bayrak et al., including 155 patients, malignancy rates of 25% and 27.6% were reported for Bethesda III and Bethesda IV nodules, respectively [23]. The observed differences may reflect variations in sample size and patient selection. Furthermore, as a major tertiary referral center, our institution receives patients from across Türkiye, contributing to the heterogeneity of the study population despite the single-center design.

It is known that repeated FNAB application is a frequently preferred approach in the Bethesda 3 group. However, it has previously been shown that the diagnostic contribution of repeated biopsies may be limited and uncertainty may persist [11,12,24]. This situation increases the need for alternative or complementary evaluation methods. In our study, the performance increase obtained by adding USG features to the model provides a solution for this need.

The role of ultrasonography in malignancy risk assessment has long been known. The strong association of findings such as hypoechogenicity, microcalcification and irregular margins with malignancy has been demonstrated in various studies [16,17,25]. TIRADS-based systems developed in line with these findings are widely used in clinical practice [18,19,21]. Nevertheless, it has also been reported that the performance of these systems may be limited, especially in indeterminate nodules [20]. In our study, the limited performance of USG findings alone is consistent with this literature.

The identification of microcalcification as the strongest independent predictor in multivariable analysis is parallel with previous studies. The strong association of microcalcifications with papillary thyroid carcinoma is well known [16,25]. Similarly, irregular margins and hypoechogenicity were also found to be independent predictors and these findings are consistent with the current literature [17]. It should also be noted that the diagnostic utility of the taller-than-wide sign may vary according to histological subtype. Previous studies have demonstrated that although this feature exhibits high specificity for malignancy overall, its discriminative value is limited in follicular and oncocytic neoplasms [26,27]. Given that Bethesda III–IV nodules are enriched for follicular-patterned lesions, omission of this parameter is unlikely to have substantially affected the overall performance of the proposed model. These results support that the selected USG parameters are appropriate for the model.

The relationship between thyroid nodule size and malignancy remains controversial and no clear consensus has been established in the literature. While several studies have suggested that larger nodules are associated with an increased risk of malignancy and reduced diagnostic accuracy of FNAB, others have demonstrated a higher malignancy risk among smaller nodules [28,29,30,31,32,33,34]. Notably, the highest malignancy rates have been reported in nodules measuring ≤2 cm [35]. In the present study, malignant nodules were significantly smaller than benign nodules (19.1 ± 7.8 mm vs. 22.4 ± 9.1 mm, *p* < 0.001). However, because thyroid nodule size is not incorporated as a malignancy-scoring parameter in commonly used ultrasonographic risk stratification systems and is primarily used to guide biopsy and management decisions, it was not included in the final multivariable model. Nevertheless, these findings suggest that nodule size alone should not be considered a reassuring feature in cytologically indeterminate thyroid nodules.

Patient age should also be considered when interpreting thyroid nodule characteristics. Previous studies have demonstrated that increasing age is positively associated with thyroid nodule formation and multinodularity [36]. In our cohort, patients with malignant nodules were slightly younger than those with benign nodules; however, given the primary objective of evaluating ultrasonographic predictors, age was not incorporated into the final multivariable model.

One of the most important findings of our study is the marked increase obtained in diagnostic performance with the combined use of cytology and ultrasonography. While the AUC values were 0.60 and 0.61 when FNAB and USG were evaluated alone, respectively, the increase in this value to 0.78 in the combined model is noteworthy. Although there are studies in the literature similarly reporting that multimodal approaches provide performance improvement, studies in which this increase is quantitatively demonstrated through model comparison are limited [11,22]. In this respect, our study provides an important contribution to the literature.

In the model comparison analysis, the observation of a significant decrease in the −2 log likelihood value and an increase in the AUC value with the addition of USG findings demonstrates that ultrasonography provides an independent contribution to cytology. This finding reveals that ultrasonography is not only a supportive method but also a component playing an active role in the decision-making process.

The results obtained in subgroup analyses are also noteworthy. Particularly in the Bethesda III group, the high negative predictive value (93.8%) may be considered an important parameter in avoiding surgery in low-risk patients. Similarly, the high AUC value obtained in the Bethesda IV group demonstrates that high-risk patients may be classified more accurately. These findings reveal that the model shows consistent performance across different risk groups.

Notably, the combined model demonstrated a negative predictive value of 93.8% in the Bethesda III subgroup, highlighting its potential role in supporting conservative management strategies in selected patients. However, these findings should be interpreted with caution until validated in prospective, multicenter studies designed to assess the model’s performance in routine clinical practice.

When the clinical implications of the study are evaluated, it is considered that the obtained findings may contribute to surgical decision-making processes. Particularly in indeterminate nodules, instead of a decision-making approach based solely on cytology, a more holistic evaluation strategy integrating USG findings may be adopted. This approach may both reduce unnecessary surgery rates and contribute to more accurate identification of malignant cases.

The study has several limitations. First, due to the retrospective design of the study, the possibility of selection bias exists and some patient-related clinical information was not consistently available in the medical records. In addition, inclusion of only surgically treated patients may have led to higher malignancy rates compared to the real population. Nevertheless, the large sample size and the presence of histopathological confirmation in all patients are among the strengths of the study. Future studies may explore the integration of clinical, biochemical and molecular variables with the present FNAB–USG model to further enhance risk stratification and personalized decision-making in patients with cytologically indeterminate thyroid nodules.

In conclusion, this study demonstrates that USG findings provide a significant additional diagnostic contribution beyond cytological evaluation in Bethesda III–IV thyroid nodules. The use of a multimodal approach may allow more accurate risk stratification, particularly in nodules with high diagnostic uncertainty.

## 5. Conclusions

In this study, it was demonstrated that the addition of USG findings to FNAB significantly increased diagnostic performance in Bethesda category III–IV thyroid nodules. In particular, the higher discriminative power and negative predictive value of the combined model reveal the limitations of cytology-based standalone evaluation approaches. The use of USG features together with cytological data may contribute to the surgical decision-making process by improving clinical risk stratification. These findings support the importance of multimodal evaluation, particularly in Bethesda III–IV nodules with high diagnostic uncertainty.

## Figures and Tables

**Table 1:** Baseline clinical and USG characteristics according to final histopathology.

Variable	Overall (*n* = 1800)	Benign (*n* = 1350)	Malignant (*n* = 450)	*p* Value
Age (years), mean ± SD	46.8 ± 12.4	47.3 ± 12.6	45.2 ± 11.9	0.002
Female sex, *n* (%)	1328 (73.8)	1005 (74.4)	323 (71.8)	0.28
Nodule size (mm), mean ± SD	21.6 ± 8.9	22.4 ± 9.1	19.1 ± 7.8	<0.001
Bethesda III, *n* (%)	1223 (67.9)	989 (73.3)	234 (52.0)	<0.001
Bethesda IV, *n* (%)	577 (32.1)	361 (26.7)	216 (48.0)	<0.001
Hypoechogenicity, *n* (%)	769 (42.7)	508 (37.6)	261 (58.0)	<0.001
Microcalcification, *n* (%)	643 (35.7)	371 (27.5)	272 (60.4)	<0.001
Irregular margins, *n* (%)	724 (40.2)	452 (33.5)	272 (60.4)	<0.001

Continuous variables are presented as mean ± standard deviation and compared using the independent samples *t*-test. Categorical variables are expressed as number (percentage) and compared using the chi-square test.

**Table 2:** Malignancy rates according to Bethesda category (III–IV).

FNAB Category	Total (*n* = 1800)	Malignant *n* (%)	Benign *n* (%)	Malignancy Rate (%)	*p* Value
Bethesda III	1223	234 (19.1)	989 (80.9)	19.1	<0.001
Bethesda IV	577	216 (37.4)	361 (62.6)	37.4	<0.001

Malignancy rates were compared using the chi-square test. Percentages represent row percentages within each Bethesda category.

**Table 3:** Univariable logistic regression analysis of USG features associated with malignancy.

Variable	Malignant *n* (%)	Benign *n* (%)	Odds Ratio (OR)	95% CI	*p* Value
Hypoechogenicity	261 (58.0)	508 (37.6)	2.30	1.87–2.83	<0.001
Microcalcification	272 (60.4)	371 (27.5)	3.99	3.23–4.93	<0.001
Irregular margins	272 (60.4)	452 (33.5)	3.02	2.45–3.72	<0.001

Odds ratios (ORs) were calculated using univariable logistic regression analysis with final histopathologic malignancy as the dependent variable. Confidence intervals (CIs) are reported at the 95% level.

**Table 4:** Multivariable logistic regression analysis of independent predictors of malignancy.

Variable	Adjusted OR	95% CI	*p* Value
Bethesda IV (vs. III)	2.11	1.65–2.70	<0.001
Hypoechogenicity	1.72	1.34–2.21	<0.001
Microcalcification	2.48	1.89–3.25	<0.001
Irregular margins	1.93	1.46–2.54	<0.001

Multivariable logistic regression analysis was performed including Bethesda category and USG features. Final histopathologic diagnosis of malignancy was used as the dependent variable. Adjusted odds ratios (ORs) with 95% confidence intervals (CIs) are presented.

**Table 5:** Diagnostic performance of FNAB, USG, and the combined model for predicting malignancy.

Parameter	FNAB Alone	USG Alone	Combined Model
Sensitivity (%)	76.0	58.0	82.4
Specificity (%)	43.4	62.4	70.1
Positive Predictive Value (%)	30.9	33.9	47.6
Negative Predictive Value (%)	84.4	81.7	92.1
Accuracy (%)	51.6	61.3	74.8
AUC (95% CI)	0.60 (0.57–0.63)	0.61 (0.58–0.64)	0.78 (0.75–0.81)

The combined model represents the predicted probability derived from multivariable logistic regression including Bethesda category and USG features. AUC values were calculated using ROC analysis.

**Table 6:** Diagnostic performance of the combined FNAB–USG model stratified by Bethesda category.

Parameter	Bethesda III (*n* = 1223)	Bethesda IV (*n* = 577)
Malignancy rate (%)	19.1	37.4
Sensitivity (%)	76.9	85.2
Specificity (%)	74.8	68.1
Positive Predictive Value (%)	39.8	58.3
Negative Predictive Value (%)	93.8	88.9
Accuracy (%)	75.3	73.4
AUC (95% CI)	0.74 (0.70–0.78)	0.80 (0.76–0.84)

The diagnostic performance of the combined multivariable model was evaluated separately in Bethesda III and Bethesda IV nodules. Predicted probabilities derived from logistic regression were used to construct ROC curves and calculate AUC values.

**Table 7:** Incremental predictive value of adding USG features to FNAB for predicting malignancy.

Model	−2 Log Likelihood	AIC	AUC (95% CI)	ΔAUC	*p* Value *
Model 1: FNAB alone	2018.4	2022.4	0.60 (0.57–0.63)	—	—
Model 2: FNAB + USG features	1786.2	1796.2	0.78 (0.75–0.81)	+0.18	<0.001

Model comparison was performed using likelihood ratio testing. The addition of USG features significantly improved model fit (Δ−2 Log Likelihood = 232.2, *p* < 0.001). Discriminative performance was assessed using ROC analysis. ΔAUC represents the difference in area under the curve between the two models. * *p* value derived from likelihood ratio test comparing nested models.

## Data Availability

The datasets generated and/or analyzed during the current study are not publicly available due to institutional and ethical restrictions but are available from the corresponding author on reasonable request.

## References

[1] Dean D.S., Gharib H. (2008). Epidemiology of thyroid nodules. Best Pract. Res. Clin. Endocrinol. Metab..

[2] Guth S., Theune U., Aberle J., Galach A., Bamberger C.M. (2009). Very high prevalence of thyroid nodules detected by high frequency (13 MHz) ultrasound examination. Eur. J. Clin. Investig..

[3] Mu C., Ming X., Tian Y., Liu Y., Yao M., Ni Y., Liu Y., Li Z. (2022). Mapping global epidemiology of thyroid nodules among general population: A systematic review and meta-analysis. Front. Oncol..

[4] Alexander E.K., Cibas E.S. (2022). Diagnosis of thyroid nodules. Lancet Diabetes Endocrinol..

[5] Grani G., Sponziello M., Filetti S., Durante C. (2024). Thyroid nodules: Diagnosis and management. Nat. Rev. Endocrinol..

[6] Durante C., Hegedüs L., Czarniecka A., Paschke R., Russ G., Schmitt F., Soares P., Solymosi T., Papini E. (2023). 2023 European Thyroid Association Clinical Practice Guidelines for thyroid nodule management. Eur. Thyroid J..

[7] Haugen B.R., Alexander E.K., Bible K.C., Doherty G.M., Mandel S.J., Nikiforov Y.E., Pacini F., Randolph G.W., Sawka A.M., Schlumberger M. (2016). 2015 American Thyroid Association Management Guidelines for Adult Patients with Thyroid Nodules and Differentiated Thyroid Cancer. Thyroid.

[8] Ali S.Z., Baloch Z.W., Cochand-Priollet B., Schmitt F.C., Vielh P., VanderLaan P.A. (2023). The 2023 Bethesda System for Reporting Thyroid Cytopathology. Thyroid.

[9] Bongiovanni M., Spitale A., Faquin W.C., Mazzucchelli L., Baloch Z.W. (2012). The Bethesda System for Reporting Thyroid Cytopathology: A meta-analysis. Acta Cytol..

[10] Ali S.Z., VanderLaan P.A. (2023). The Bethesda System for Reporting Thyroid Cytopathology.

[11] Kim J., Shin J.H., Oh Y.L., Hahn S.Y., Park K.W. (2022). Approach to Bethesda system category III thyroid nodules according to US-risk stratification. Endocr. J..

[12] VanderLaan P.A., Marqusee E., Krane J.F. (2011). Clinical outcome for atypia of undetermined significance in thyroid fine-needle aspirations: Should repeated FNA be the preferred initial approach?. Am. J. Clin. Pathol..

[13] Faquin W.C., Wong L.Q., Afrogheh A.H., Ali S.Z., Bishop J.A., Bongiovanni M., Pusztaszeri M.P., VandenBussche C.J., Gourmaud J., Vaickus L.J. (2016). Impact of reclassifying noninvasive follicular variant of papillary thyroid carcinoma on the risk of malignancy in The Bethesda System for Reporting Thyroid Cytopathology. Cancer Cytopathol..

[14] Iskandar M.E., Bonomo G., Avadhani V., Persky M., Lucido D., Wang B., Marti J.L. (2015). Evidence for overestimation of the prevalence of malignancy in indeterminate thyroid nodules classified as Bethesda category III. Surgery.

[15] Strickland K.C., Howitt B.E., Marqusee E., Alexander E.K., Cibas E.S., Krane J.F., Barletta J.A. (2015). The impact of noninvasive follicular variant of papillary thyroid carcinoma on rates of malignancy for fine-needle aspiration diagnostic categories. Thyroid.

[16] Remonti L.R., Kramer C.K., Leitão C.B., Pinto L.C., Gross J.L. (2015). Thyroid ultrasound features and risk of carcinoma: A systematic review and meta-analysis of observational studies. Thyroid.

[17] Smith-Bindman R., Lebda P., Feldstein V.A., Sellami D., Goldstein R.B., Brasic N., Jin C., Kornak J. (2013). Risk of thyroid cancer based on thyroid ultrasound imaging characteristics: Results of a population-based study. JAMA Intern. Med..

[18] Russ G., Bonnema S.J., Erdogan M.F., Durante C., Ngu R., Leenhardt L. (2017). European Thyroid Association Guidelines for Ultrasound Malignancy Risk Stratification of Thyroid Nodules in Adults: The EU-TIRADS. Eur. Thyroid J..

[19] Tessler F.N., Middleton W.D., Grant E.G., Hoang J.K., Berland L.L., Teefey S.A., Cronan J.J., Beland M.D., Desser T.S., Frates M.C. (2017). ACR Thyroid Imaging, Reporting and Data System (TI-RADS): White Paper of the ACR TI-RADS Committee. J. Am. Coll. Radiol..

[20] Yang L., Li C., Chen Z., He S., Wang Z., Liu J. (2023). Diagnostic efficiency among EU-/C-/ACR-TIRADS and S-Detect for thyroid nodules: A systematic review and network meta-analysis. Front. Endocrinol..

[21] Yucel S., Balci I.G., Tomak L. (2023). Diagnostic performance of thyroid nodule risk stratification systems: Comparison of ACR-TIRADS, EU-TIRADS, K-TIRADS, and ATA guidelines. Ultrasound Q..

[22] Carrillo J.F., Frias-Mendivil M., Ochoa-Carrillo F.J., Ibarra M. (2000). Accuracy of fine-needle aspiration biopsy of the thyroid combined with an evaluation of clinical and radiologic factors. Otolaryngol. Head Neck Surg..

[23] Yaprak Bayrak B., Eruyar A.T. (2020). Malignancy rates for Bethesda III and IV thyroid nodules: A retrospective study of the correlation between fine-needle aspiration cytology and histopathology. BMC Endocr. Disord..

[24] Jack G.A., Sternberg S.B., Aronson M.D., Mukamal K.J., Oshin A., Hennessey J.V. (2020). Nondiagnostic fine-needle aspiration biopsy of thyroid nodules: Outcomes and determinants. Thyroid.

[25] Brito J.P., Gionfriddo M.R., Al Nofal A., Boehmer K.R., Leppin A.L., Reading C., Callstrom M., Elraiyah T.A., Prokop L.J., Stan M.N. (2014). The accuracy of thyroid nodule ultrasound to predict thyroid cancer: Systematic review and meta-analysis. J. Clin. Endocrinol. Metab..

[26] Fukushima M., Fukunari N., Murakami T., Kunii Y., Suzuki S., Kitaoka M. (2021). Reconfirmation of the accuracy of the taller-than-wide sign in multicenter collaborative research in Japan. Endocr. J..

[27] Bell C., White S.L., Tylee T., Dighe M., La Greca A., Goldner W., Mayson S., Haugen B.R., Pozdeyev N. (2025). Thyroid Nodule Sphericity Metrics Discriminate Benign and Malignant Follicular and Oncocytic Neoplasms. Thyroid.

[28] Koo D.H., Song K., Kwon H., Bae D.S., Kim J.-H., Min H.S., Lee K.E., Youn Y.-K. (2016). Does Tumor Size Influence the Diagnostic Accuracy of Ultrasound-Guided Fine-Needle Aspiration Cytology for Thyroid Nodules?. Int. J. Endocrinol..

[29] Mehanna R., Murphy M., McCarthy J., O’Leary G., Tuthill A., Murphy M.S., Sheahan P. (2013). False negatives in thyroid cytology: Impact of large nodule size and follicular variant of papillary carcinoma. Laryngoscope.

[30] McCoy K.L., Jabbour N., Ogilvie J.B., Ohori N.P., Carty S.E., Yim J.H. (2007). The incidence of cancer and rate of false-negative cytology in thyroid nodules greater than or equal to 4 cm in size. Surgery.

[31] Magister M.J., Chaikhoutdinov I., Schaefer E., Williams N., Saunders B., Goldenberg D. (2015). Association of Thyroid Nodule Size and Bethesda Class with Rate of Malignant Disease. JAMA Otolaryngol. Head Neck Surg..

[32] Ucler R., Usluogulları C.A., Tam A.A., Ozdemir D., Balkan F., Yalcın S., Kıyak G., Ersoy P.E., Guler G., Ersoy R. (2015). The diagnostic accuracy of ultrasound-guided fine-needle aspiration biopsy for thyroid nodules three centimeters or larger in size. Diagn. Cytopathol..

[33] Shrestha M., Crothers B.A., Burch H.B. (2012). The impact of thyroid nodule size on the risk of malignancy and accuracy of fine-needle aspiration: A 10-year study from a single institution. Thyroid.

[34] Kamran S.C., Marqusee E., Kim M.I., Frates M.C., Ritner J., Peters H., Benson C.B., Doubilet P.M., Cibas E.S., Barletta J. (2013). Thyroid nodule size and prediction of cancer. J. Clin. Endocrinol. Metab..

[35] Cavallo A., Johnson D.N., White M.G., Siddiqui S., Antic T., Mathew M., Grogan R.H., Angelos P., Kaplan E.L., Cipriani N.A. (2017). Thyroid Nodule Size at Ultrasound as a Predictor of Malignancy and Final Pathologic Size. Thyroid.

[36] Kwong N., Medici M., Angell T.E., Liu X., Marqusee E., Cibas E.S., Krane J.F., Barletta J.A., Kim M.I., Larsen P.R. (2015). The Influence of Patient Age on Thyroid Nodule Formation, Multinodularity, and Thyroid Cancer Risk. J. Clin. Endocrinol. Metab..

